# The efficacy and safety of tranexamic acid in revision total knee arthroplasty: a meta-analysis

**DOI:** 10.1186/s12891-017-1633-y

**Published:** 2017-06-21

**Authors:** Peng Tian, Wen-bin Liu, Zhi-jun Li, Gui-jun Xu, Yu-ting Huang, Xin-long Ma

**Affiliations:** 1 Department of Orthopedics, Tianjin Hospital, No. 406, Jiefang Nan Road, Tianjin, 300211 People’s Republic of China; 2 Department of Joint Surgery, Tianjin Hospital, No. 406, Jiefang Nan Road, Tianjin, 300211 People’s Republic of China; 3 Department of Orthopedics, General Hospital of Tianjin Medical University, No. 154, Anshan Road, Tianjin, 300052 People’s Republic of China; 4 Cancer & Immunology Research, Children’s Research Institute, Children’s National Medical Center, 111 Michigan Avenue, NW, Washington, DC 20010 USA

**Keywords:** Knee, Arthroplasty, Revision, Tranexamic acid, Meta-analysis

## Abstract

**Background:**

There is no consistent conclusion regarding the efficacy and safety of the intravenous administration of tranexamic acid (TXA) for reducing blood loss in revision total knee arthroplasty (TKA). We performed a meta-analysis of comparative trials to evaluate the efficacy and safety of TXA in revision TKA.

**Methods:**

We conducted a search of PubMed, EMBASE, The Cochrane Library and Web of Science for randomized controlled trials (RCTs) and non-RCTs. Two authors selected the studies, extracted the data, and assessed the risk of bias independently. A pooled meta-analysis was performed using RevMan 5.3 software.

**Results:**

Four non-RCTs met the inclusion criteria. The meta-analysis indicated that the use of TXA was related to significantly less transfusion requirements (RD = −0.25; 95% CI: -0.43 to −0.08; *P* = 0.005), drainage volume (MD = −321.07; 95% CI: -445.13 to −197.01, *P* = 0.005), hemoglobin reduction (MD = −0.52; 95% CI: -0.79 to −0.25, *P* = 0.0001), and length of hospital stay (MD = −2.36; 95% CI: -4.00 to −0.71, *P* = 0.005). No significant differences in the incidence of deep venous thrombosis (DVT) or pulmonary embolism (PE) were noted.

**Conclusions:**

The use of TXA for patients undergoing revision TKA may reduce blood loss and transfusion requirements without increasing the risk of postoperative venous thromboembolism. Due to the limited quality of the currently available evidence, more high-quality RCTs are required.

## Background

Total knee arthroplasty (TKA) is a common surgical method for the treatment of end-stage knee disease, which could effectively relieve knee pain and greatly improve patient quality of life [1, 2]. A substantial increase in the prevalence of TKA over time and a shift to younger ages was noted [3], such that the number of revision TKAs has increased annually [4]. The common causes of revision TKA include infection, mechanical loosening, and pain and knee instability [5]. Compared with primary TKA, revision TKA may be challenging due to longer operative time and more blood loss [6]. Controlling blood loss and reducing transfusion rates are problems that clinical orthopedic surgeons face.

Tranexamic acid (TXA) has been widely used to reduce blood loss and transfusion requirements in primary TKA [7–9]. However, little is known about the efficacy and safety of the use of TXA in revision TKA. Recently, several published studies have demonstrated that TXA could safely and effectively reduce blood loss and transfusion rates in revision TKA [10–13]. However, some of these studies have been criticized for poor design, low power, inconclusive results and small sample size. It is imperative to clarify whether the use of TXA is effective in revision TKA. Thus, we conducted a meta-analysis to ascertain whether the application of TXA would reduce blood loss and transfusion requirements in revision TKA.

## Methods

This meta-analysis was performed in accordance with the Preferred Reporting Items for Systematic Reviews and Meta-Analyses reporting guidelines for the meta-analysis of intervention trials.

### Inclusion and exclusion criteria

Studies were included if the following criteria were met: (1) study design: comparative studies (randomized controlled trials, RCTs or non-RCTs); (2) study subjects: adult patients with indications for revision TKA; (3) operative intervention: patients in the TXA group received intravenous TXA and patients in the control group received placebo or nothing; (4) outcome measures: the primary outcomes included calculated total blood loss, hidden blood loss, transfusion rate, and postoperative complications. Secondary outcomes included hemoglobin reduction, surgical duration, and length of hospital stay.

Articles that reported at least one outcome were included, and those without the outcome measures of interest were excluded. Letters, comments, editorials, reviews and practice guidelines were excluded. Any controversy was cross-checked and resolved by a third author to reach a final consensus.

### Search strategy

PubMed, Medline, Embase, Web of Science and the Cochrane Library were searched up to October 2016 for comparative studies involving TXA for reducing blood loss in patients undergoing revision TKA. The search terms were as follows: “tranexamic acid”, “knee arthroplasty”, “knee replacement” and “revision”. The language for the publications was limited to English. The titles and abstracts of studies identified in the search were reviewed to exclude clearly irrelevant studies. The reference lists of all eligible studies and relevant reviews were searched manually for additional trials. The search for titles and abstracts was conducted independently by two reviewers. Disagreements were resolved by consulting a third reviewer.

### Quality assessment

Two authors independently assessed the risk of bias of the included studies. RCTs were assessed with the RCT bias risk assessment tools of the Cochrane Handbook Version 5.3 [14]. Non-RCTs were assessed with the Methodological Index for Non-randomized Studies (MINORS) [15]. Disagreement was resolved by the third author.

### Data extraction

For each eligible study, both reviewers extracted all the relevant data independently. Data available in articles or tracked by e-mail were extracted independently by two authors. The following variables were recorded: name of the first author, year of publication, sample size, participant sex and age, revision reason, surgical approach, anesthesia and outcome measurements. Data in other forms (i.e., median, interquartile range, and mean ± 95% confidence interval (CI)) were converted to mean ± standard deviation (SD) according to the Cochrane Handbook. If data were not reported numerically, we extracted them by manual measurements from published figures.

### Data analysis and statistical methods

All statistical analyses were performed with Review Manager (version 5.3 for Windows, The Cochrane Collaboration, The Nordic Cochrane Centre, Copenhagen, 2008). The mean difference (MD) with a 95% CI was calculated for continuous data. The risk difference (RD) with 95% CI was calculated for dichotomous data. Heterogeneity among studies was estimated using the I^2^ statistic; substantial heterogeneity was represented by I^2^ > 50%. A random effects model was used if the heterogeneity test was not significant (I^2^ > 50%; *P* < 0.1). Otherwise, we adopted the fixed-effects model, and *P* < 0.05 was considered significant. Subgroup analysis was performed to explore the impact of an individual study by removing one study from the analysis each time.

## Results

### Study characteristics

**Fig. 1:**
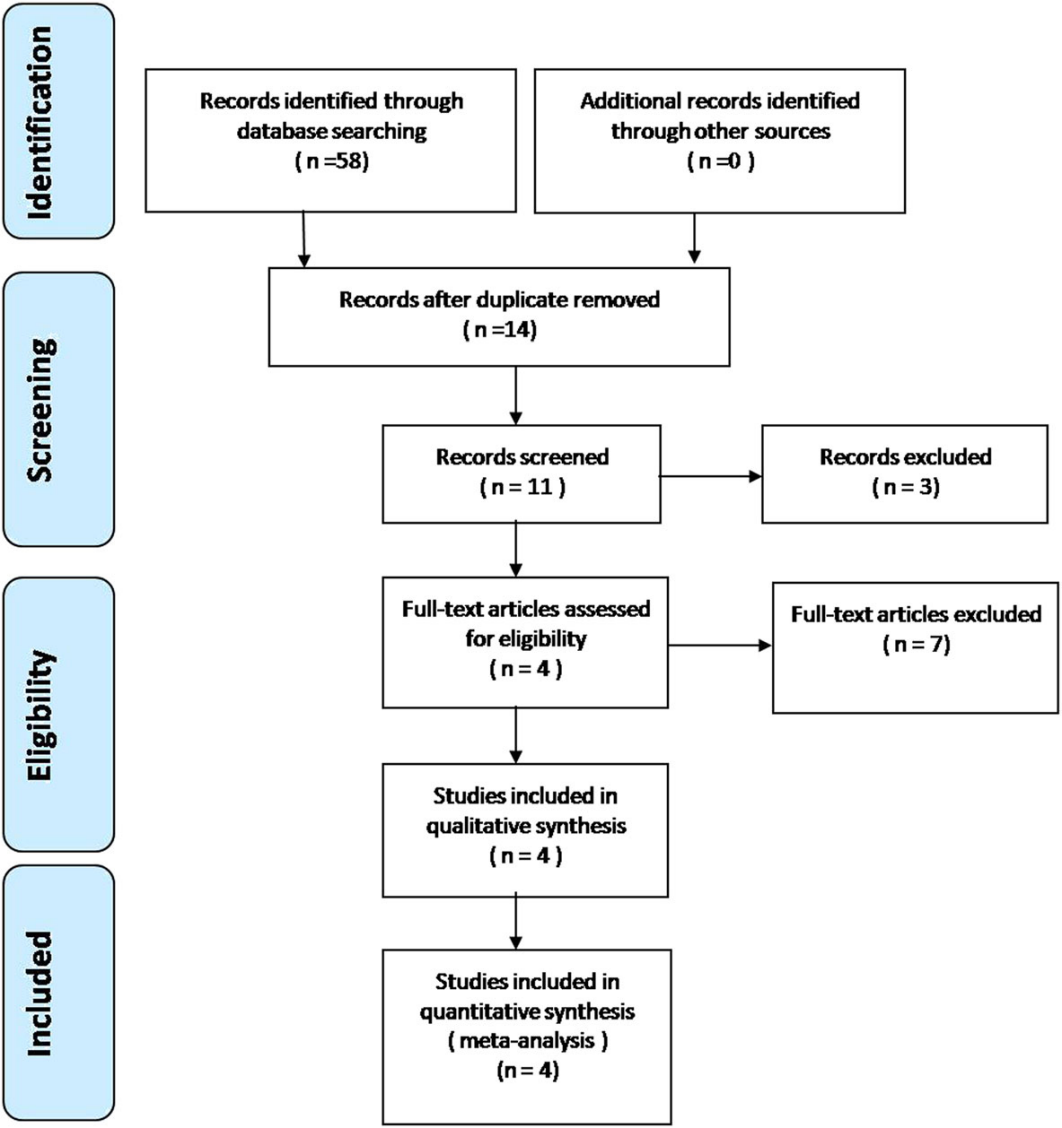
Flowchart of the study selection process

The search process is shown in Fig. 1. Table 1 summarizes the characteristics of the four included studies, which were published between 2012 and 2016. The studies’ sample size was 47–422 patients. All of the trials involved revision TKA. Baseline characteristics between the two groups in each study were well matched. All included studies reported the use of a tourniquet and chemoprophylaxis for deep venous thrombosis (DVT) or pulmonary embolism (PE). All included studies reported no differences in preoperative hemoglobin between the two groups. All studies described an indication for transfusion associated with a reduction in hemoglobin level or hematocrit and clinical symptoms. Thromboembolic complications, such as DVT or PE, were reported in three studies [10, 12, 13]. Two included studies reported that revision components were implanted in both the femur and the tibia [10, 11]. The other two studies stated patients were undergoing revision of two components, revision of one component, or an isolated liner exchange.

**Table 1:** Characteristics of included studies

Study	Group	Simple size	Age (Y)	Gender (M/F)	Preoperative hemoglobin (g/l)	Intervention	Diagnosis
Aguilera 2012 [10]	TXA	19	75	2/17	132	1 g	Aseptic loosening
	Control	28	74	9/19	134		
Ortega-Andreu 2016 [11]	TXA	44	68.8	12/32	141	two doses of 15 mg/kg per dose	Aseptic loosening; Pain; Polyethylene wear; Prosthesis malposition; Fracture
	Control	43	74.2	11/32	138		
Samujh 2014 [12]	TXA	43	65.4	14/19	132	15 mg/Kg	NS
	Control	68	64.7	29/39	135		
Smit 2013 [13]	TXA	246	68.9	121/125	132.1	20 mg/Kg	Loosening; Infection; Polywear; Instability; Stiffness; Malalignment; Patellar problems; Pain
	Control	178	69.8	87/91	129.9		

### Risk of bias assessment

Four included studies were non-RCTs, and the MINORS scores were 18–20 for the retrospectively controlled trials. The methodological quality assessment is presented in Table 2.

**Table 2:** Quality assessment for non-randomized trials

Quality assessment for non-randomized trials	Aguilera 2012 [10]	Ortega-Andreu 2016 [11]	Samujh 2014 [12]	Smit 2013 [13]
A clearly stated aim	2	2	2	2
Inclusion of consecutive patients	2	2	2	2
Prospective data collection	2	2	2	2
Endpoints appropriate to the aim of the study	2	2	2	2
Unbiased assessment of the study endpoint	0	0	0	0
A follow-up period appropriate to theaims of study	2	2	2	2
Less than 5% loss to follow-up	2	2	2	2
Prospective calculation of the sample size	0	0	0	0
An adequate control group	2	2	2	2
Contemporary groups	0	2	1	2
Baseline equivalence of groups	2	2	2	2
Adequate statistical analyses	2	2	2	2
Total score	18	20	19	20

### Outcomes of the meta-analysis

#### Transfusion requirements

Four studies involving 667 patients were used to perform a meta-analysis on the requirements of blood transfusion [10–13]. The transfusion rate was 14.5% and 33.8% in the TXA (51 patients) and control groups (107 patients), respectively. Significant heterogeneity was observed, and a random effects model was used (I^2^ = 85%, *P* = 0.0002). The meta-analysis revealed significant differences in transfusion requirements between the two groups (RD = −0.25; 95% CI: -0.43 to −0.08; *P* = 0.005, Table 3).

**Table 3:** Meta-analysis results

Outcome	Studies	Groups (TXA/Placebo)	Overall effect			Heterogeneity	
			Effect estimate	95% CI	*p*-Value	I^2^(%)	*p*-Value
Transfusion requirements	4	352/317	−0.25	−0.43, −0.08	0.005	85	0.0002
Drainage volume	2	63/72	−321.07	−445.13, −197.01	0.005	0	0.68
Hb reduction	2	290/221	−0.52	−0.79, −0.25	0.0001	62	0.11
Length of hospital stay	3	309/249	−2.36	−4.00, −0.71	0.005	70	0.04
Deep venous thrombosis	3	308/274	0.00	−0.01, 0.02	0.69	0	0.97
Pulmonary embolism	2	289/246	−0.01	−0.03, 0.01	0.18	0	0.89

*TXA* tranexamic acid, *CI* confidence interval

#### Drainage volume

Data from two studies involving 135 patients were available to examine drainage volume [10, 11]. No significant heterogeneity was observed, and a fixed effects model was used (I^2^ = 0%, *P* = 0.68). The application of TXA in revision TKA produced significantly less drainage volume compared with the control group (MD = −321.07; 95% CI: -445.13 to −197.01, *P* = 0.005, Table 3).

#### Hemoglobin reduction

Two studies involving 511 patients reported hemoglobin reduction after revision TKA [11, 13]. No significant heterogeneity was observed, and a fixed effects model was used (I^2^ = 62%, *P* = 0.11). A significant difference was observed between the two groups regarding the amount of hemoglobin reduction after revision TKA (MD = −0.52; 95% CI: -0.79 to −0.25, *P* = 0.0001, Table 3).

#### Length of hospital stay

Data were available from three studies involving 558 patients [10, 11, 13]. Significant heterogeneity was observed, and a random effects model was used (I^2^ = 70%, *P* = 0.04). A statistically significant difference in the length of hospital stay was noted between the two groups (MD = −2.36; 95% CI: -4.00 to −0.71, *P* = 0.005, Table 3).

#### Deep venous thrombosis

Three studies reported the post-operative incidence of DVT [10, 12, 13]. No significant heterogeneity was observed, and a fixed effects model was used (I^2^ = 0%, *P* = 0.97). The meta-analysis revealed no significant difference in the post-operative incidence of DVT between the two groups (MD = 0.00, 95% CI: -0.01 to 0.02, *P* = 0.69, Table 3).

#### Pulmonary embolism

Two studies reported the post-operative incidence of PE [12, 13]. No significant heterogeneity was observed, and a fixed effects model was used (I^2^ = 0%, *P* = 0.89). The meta-analysis revealed no significant difference in the post-operative incidence of PE between two groups (MD = −0.01, 95% CI: -0.03 to 0.01, *P* = 0.18, Table 3).

## Discussion

Although the patients with TKA can experience good functional outcomes and long-term implant survivorship [2], TKA failure and revision TKA continue to be a significant clinical challenge for orthopedic surgeons. Revision TKA could produce more blood loss and higher transfusion rates compared with primary TKA [16]. Perioperative blood loss is an inevitable complication of revision TKA, which could lead to anemia. Effective blood management can minimize blood loss and transfusions such that patients achieve better results. TXA, an antifibrinolytic agent, could be effective and safe in reducing blood loss in primary TKA. The main applications of TXA are intravenous or intra-articular injection. However, there is relatively little research on the role and dosing regimen of TXA in revision TKA.

The most important findings of the present meta-analysis are that the application of intravenous TXA for patients undergoing revision TKA may reduce the transfusion rate, drainage volume, hemoglobin reduction and length of hospital stay without increasing the risks of DVT or PE. To our knowledge, this is the first meta-analysis of comparative trials to evaluate the efficacy and safety of TXA in revision TKA.

To date, the efficacy and safety of intravenous TXA for reducing blood loss and transfusion rates in revision TKA remains controversial. Many scholars continue to explore the strategy of intravenous TXA administration in revision TKA. In revision TKA, preoperative hemoglobin could be an important factor to avoid transfusion. All included studies indicated no differences from preoperative hemoglobin between two groups. The present meta-analysis demonstrated that hemoglobin reduction was reduced in the TXA group compared with the control group.

The pooled results demonstrated significant differences in the transfusion rate between the TXA group and the control group. The transfusion rate was 14.5% and 33.8% in the TXA group and the control group, respectively. Aguilera et al. [10] first evaluated the effectiveness of TXA in revision TKA and provided evidence that the early administration of TXA could decrease the transfusion rate of the patients with revision TKA. Ortega-Andreu et al. [11] confirmed that a two-dose intravenous administration of TXA in revision TKA was effective in decreasing hemoglobin loss and the transfusion rate. Although all included studies described an indication for transfusion associated with a reduction in hemoglobin level or hematocrit and clinical symptoms, the outcome of units transfused was reported in two studies that were not analyzed owing to insufficient data.

The application of TXA could reduce postoperative drainage volume and earlier drainage tube removal, which could be helpful for rapid recovery [11]. TXA could also be associated with shortening the length of hospital stay [13]. The present meta-analysis revealed that the application of TXA could significantly reduce the average length of hospital stay and drainage in patients undergoing revision TKA. Reducing transfusion rates and minimizing the average length of hospital stay could reduce financial burden. According to the cost savings calculation [17], there would be a potential yearly cost savings of $22,300 with the application of TXA in revision TKA [13].

The application of TXA did not increase the incidence of DVT and PE in patients undergoing primary TKA. Therefore, the application of TXA is mainly concerned about the incidence of thromboembolic events in revision TKA. In the present meta-analysis, no significant differences in the incidence of DVT or PE were noted between the TXA group and the control group.

It is imperative to acknowledge some potential limitations in our meta-analysis: (1) the sample sizes of the included studies were relatively small, (2) the methodologies of the included studies have their own limitations, and (3) there were some differences in TXA and dosing regimen. Given the above defects and deficiencies, the pooled estimates should be explored with caution.

## Conclusion

The application of intravenous TXA for patients undergoing revision TKA may reduce transfusion rate, drainage volume, hemoglobin reduction and length of hospital stay without increasing the risks of DVT or PE. Due to the limited quality and data from the studies currently available, more high-quality randomized controlled trials are required.

## Abbreviations

CI: Confidence intervals
DVT: Deep venous thrombosis
MD: Mean difference
MINORS: Methodological Index for Non-randomized Studies
PE: Pulmonary embolism
RD: Risk difference
SD: Standard deviation
TKA: Total knee arthroplasty
TXA: Tranexamic acid
